# Audience-response systems for evaluation of pediatric lectures – comparison with a classic end-of-term online-based evaluation

**DOI:** 10.3205/zma000960

**Published:** 2015-05-13

**Authors:** Sebastian Felix Nepomuk Bode, Christine Straub, Marianne Giesler, Silke Biller, Johannes Forster, Marcus Krüger

**Affiliations:** 1 Universitätsklinikum Freiburg, Zentrum für Kinder- und Jugendmedizin, Freiburg, Germany; 2 Universität Freiburg, Kompetenzzentrum Evaluation Baden-Württemberg, Freiburg, Germany; 3 Universität Basel, Medizinische Fakultät, Studiendekanat, Basel, Switzerland; 4 St. Josefskrankenhaus, Freiburg, Germany

**Keywords:** Audience Response System, ARS, Evaluation, Pediatrics, Lecture

## Abstract

**Aim: **Course evaluations are often conducted and analyzed well after the course has taken place. By using a digital audience response system (ARS), it is possible to collect, view and discuss feedback during or directly following a course or lecture session. This paper analyzes a student evaluation of a lecture course with ARS to determine if significant differences exist between the results of the ARS lecture evaluation and those of the online evaluation at the end of the semester. In terms of the overall evaluation, consideration is given to the level of students’ prior knowledge, the presentation of the lecture material by the lecturers and the relevance of the lecture topic for students.

**Method: **During the 2011-12 winter semester, the lecture on Pediatrics at the Freiburg Center for Pediatric and Adolescent Medicine (*Zentrum für Kinder- und Jugendmedizin (ZKJ) Freiburg)* was evaluated using ARS. Thirty-four lectures were evaluated by an average of 22 (range 8-44) students, who responded to four questions each time an evaluation took place.

**Results:** On a 6-point Likert scale (1=very good to 6=deficient), the students rated their level of preparedness with a mean of 3.18, the presentation of the lecture with 2.44, and the relevance of the lecture topic with 2.19. The overall evaluation of the lecture course by means of ARS resulted in 2.31. The online evaluation conducted at the end of the semester yielded a score of 2.45. Highly significant correlations were seen between the results of the ARS for the overall evaluation, assessment of prior knowledge, lecture presentation, and the estimated relevance of the lecture topic.

**Conclusion: **The use of ARS is suitable for immediate evaluation of lectures, in particular regarding timely feedback for the individual lecturerlecturers. In comparison with an end-of-term evaluation, ARS yielded a better assessment.

## Zusammenfassung

**Zielsetzung:** Lehrveranstaltungsevaluationen werden oft zeitverzögert zur Lehrveranstaltung durchgeführt und ausgewertet. Mit dem elektronischen Evaluationsmedium Audience Response System (ARS) kann unmittelbar während oder nach Ende einer Lehrveranstaltung Feedback eingeholt, dargestellt und diskutiert werden. In dieser Arbeit wird eine studentische ARS-Vorlesungsevaluation analysiert. Es wird überprüft, ob signifikante Unterschiede zwischen den Ergebnissen der ARS-Vorlesungsevaluation und der Online-Evaluation am Ende des Semesters bestehen. Hierbei wird der Zusammenhang des studentischen Vorwissens, der Gestaltung der Lehrveranstaltung durch die Lehrenden sowie der Relevanz des Vorlesungsthemas für die Studierenden in Bezug auf die Gesamtbewertung betrachtet.

**Methodik:** Im Wintersemester 2011/2012 wurde die Pädiatrievorlesung am Zentrum für Kinder- und Jugendmedizin (ZKJ) Freiburg mit Hilfe eines ARS evaluiert. Es wurden 34 Vorlesungen von durchschnittlich 22 (range 8-44) Studierenden mit jeweils vier Fragen evaluiert.

**Ergebnisse:** Auf einer 6-stufigen Likert-Skala (1=sehr gut bis 6=ungenügend) bewerteten die Studierenden ihr Vorwissen im Mittel mit 3,18, die Vorlesungsgestaltung mit 2,44 und die Relevanz des Vorlesungsthemas mit 2,19. Die Gesamtbewertung der Vorlesung mittels ARS-Evaluation ergab die Note 2,31. In der Online Evaluation am Ende des Semesters wurde die Note 2,45 ermittelt. Es zeigt sich hoch signifikante Zusammenhänge zwischen den durch den Einsatz des ARS erhobenen Ergebnissen der Gesamtbewertung, dem Vorwissen der Studierenden, der Vorlesungsgestaltung sowie der selbsteingeschätzten Relevanz des Vorlesungsthemas.

**Schlussfolgerung:** Der Einsatz von ARS ist zur unmittelbaren Vorlesungs- Evaluation, insbesondere im Hinblick auf zeitnahe (lehr-)personenbezogene Rückmeldungen geeignet. Im Vergleich zu einer Semesterende-Evaluation zeigt sich in der ARS-Evaluation eine bessere Bewertung.

## 1. Background

In Germany the medical licensing regulations (§ 2 subs. 9 ÄAppO) stipulate that educational courses must be regularly evaluated. The state law governing higher education in Baden Württemberg (§ 5 subs. 2) provides for student participation. Student evaluation of university courses is well established at medical schools and is considered reliable under certain conditions [1], [2]. Student evaluations are viewed as reliable particularly when they focus on individual courses and the lecturers receive direct feedback, for instance within the context of an lecturer coaching session [3], [4]. Both the delayed evaluation at semester’s end and the general evaluation of a lecture course only offer limited assistance for improving teaching, especially if, as is the case at the Freiburg Center for Pediatric and Adolescent Medicine (ZKJ), different areas of Pediatrics are covered by different lecturers, or the same ones are covered by different lecturers. The question arises to what extent any evaluation results gathered under these conditions are valid, and if they can be used to improve the lecture course.

The goal is to have an evaluation instrument that, with reasonable effort on the part of lecturers and students, can provide an lecturer with reliable and valid information in a timely manner concerning the quality of his or her teaching and indicate any need for improvement. Audience response systems (ARS) can provide timely information on the quality of courses, and targeted use of this still new evaluation instrument can at least partially counteract student weariness regarding evaluations [5]. The use of ARS in lecture sessions leads to higher levels of student participation and alertness [5], [6], [7], [8]. However, there is little data currently available on this form of course evaluation [7], [9], [10]. Lecturers see the rapid availability of evaluation results as the primary advantage [7], [9]. One effect of ARS, which must be viewed critically, is that the evaluation of lectures and lecturers is more positive when compared to other forms of evaluation [9], [10], [11], [12].

## 2. Aim of the study

Previously, the lecture in Pediatrics at the ZKJ was evaluated at the end of the semester using an online-based tool. A individual critique of the teaching by different lecturers did not take place. The lecturers criticized this lack of direct feedback on their own lectures in regard to content and teaching.

The aim of this investigation was to compare the results of the end-of-semester evaluation with the overall evaluation conducted with ARS. Using the variables “student preparation”, “presentation of lecture”, and “relevance of the lecture topic to students”, factors having a potential influence on the evaluation results were also to be investigated.

## 3. Method

### 3.1 Procedure

During the 2011-12 winter semester all of the lectures on Pediatrics at the ZKJ were evaluated using ARS. All of the attending students were given a device known as a “clicker” at the beginning of the lecture session. No verification was done to ascertain if all students actually did respond. The evaluation took place immediately after the lecture using the PowerVote^©^ ARS voting system (La Générale Multimédia, Clichy, France). Four questions were posed:

1. At the start of this lecture, I was already prepared for the topic;
2. The way in which the lecture is presented (language, media, speed) helps me understand the content;
3. The lecture content is probably relevant for later professional practice (even for non-pediatricians);
4. My overall evaluation of the lecture is....

Two questions addressed the quality of teaching (questions 2 and 4). Two questions were selected to gauge the extent to which the self-assessed relevance of the topic and the self-assessed level of advance preparation correlate with the presentation and overall assessment of the lecture (questions 1 and 3).

The evaluation was done using a 6-point Likert scale (1=very good to 6=deficient). By asking these questions, the lecturers were to be given direct feedback at the end of each lecture session. At the end of the semester all of the lecturers received not only the results for their own lectures, but also the mean values of all evaluations for comparison.

Since lecturers often attribute – primarily negative – student evaluations of their teaching to factors outside their influence, we looked to see if the overall assessment (question 4) correlated with student preparation (question 1) and the relevance of the lecture (question 3). In addition, the variable of content presentation was included as a control variable. The evaluations of this variable have to be more closely connected to the overall assessment than the evaluations of the other variables [13]. The score for the overall assessment from the ARS was compared with the score from the online, end-of-semester evaluation, which was conducted four months after the end of the first block in Pediatrics. We postulated that, as shown in prior studies [9], [10], [11], [12], a better evaluation result would be seen for the ARS when compared to the end-of-term evaluation.

#### 3.2 Statistical analysis

The ARS evaluation was analyzed with IBM SPSS Version 20 (IBM SPSS Statistics for Windows, version 20.0. Armonk, NY: IBM Corp.). The lecturers were assigned numerical codes to guarantee anonymity during analysis and dissemination of the results. Correlations were calculated based on Pearson, and t-tests were carried out. To determine the effect size of the mean differences, Cohen’s d was calculated. Due to unavailable raw data for the end-of-term evaluation, the mean values of the ARS overall assessment and the mean values for the end-of-term evaluation were compared using a one-sample t-test.

## 4. Results

The lecture topics covered the areas of specialized general pediatrics, pediatric cardiology, pediatric neurology, and pediatric hematology and oncology. During the 2011-12 winter semester, the lectures on Pediatrics at the ZKJ were held in two instructional blocks of 17 lectures each. The titles of the lectures and the material covered were identical for both blocks, as was the four-week span over which the lectures took place. Eighty students in their 8^th^ semester of study participated in each block. Attendence was not compulsory and the number of participants was not counted. Twenty-one lecturers took part in the lectures.

A total of 833 “responses” were submitted by means of ARS. For each question, the mean number of “responses” given per lecture was 22 (SD=7.98; min. 8 - max. 44). The results for the four questions are presented in Table 1 (Tab. 1). In Block 1, 343 - 370 “responses” were given per question; in Block 2 the number was 405 - 463. Ninety-seven students (61% response rate) evaluated the 2011-12 winter semester pediatric lecture course using the end-of-semester evaluation online. There is a significant difference between the overall evaluation using ARS and the overall evaluation of the end-of-semester evaluation (M_ARS_=2.31, SD=1.178; M_SemEnd_=2.45; t value -3.557, df 787, p<.0001).

The results of the ARS evaluations for both lecture blocks are illustrated in Table 2 (Tab. 2). For all of the questions there are significant mean differences, with the effect sizes (Cohen’s d) lying in the lower to middle range. The results for Block 2 concerning lecture presentation are less favorable than those for Block 1. The students who attended the Block 2 lectures reported that they were less prepared in comparison with the students in Block 1. Furthermore, they estimated the relevance of the course topic to be less important than their peers in Block 1. Since it was very striking that in both blocks the range of ratings varied for the individual lecturers regarding lecture presentation (Block 1: M=1.46 to M=3.10; Block 2: M=2.00 to M=4.10) and the overall evaluation (Block 1: M=1.33 to M=3.38, Block 2: M=1.91 to M=4.43), additional analyses (not presented here) were performed separately for each block using the factor “lecturer”. The results of these analyses revealed that the mean evaluations in Block 2 for the two most poorly rated lecturers differed significantly from the mean values for the evaluations of all the other lecturers, while in Block 1 significant evaluation differences were more frequently seen between the lecturers in this same lower range. This means that the evaluations of the lecturers in Block 1 are better on the whole, but more heterogeneous. The results of the intercorrelations between the evaluations gathered through ARS are presented in Table 3 (Tab. 3). The assessments regarding lecture presentation and the topic’s relevance correlate most strongly with the overall evaluation of the course. The lecture presentation and course relevance correlate with each other in a highly significant manner.

## 5. Discussion

### 5.1 ARS participation

The use of ARS in the lecture setting is rated positively by both students and teachers [5], [6], [7], [8], [9]. No statement about the acceptance of the system can be made based on this study, since this aspect was not specifically investigated. However, oral feedback from students and lecturers was consistently positive. The ratio of students evaluating via ARS to all students present was not documented. However, all students did receive a clicker and were requested multiple times during the course to make use of it. In addition, the clicker was used as a teaching tool during the lectures.

For courses not requiring attendance, an absence rate of 18.5-70% is described [14], [15], [16]. If the number of votes cast is taken to be the actual number of students present, then the average rate of attendance (with a mean of 22 votes per question) is in the lower range of the percentages reported in the relevant studies. Should this study be repeated, documenting the number of students present will be necessary for better assessment of the results.

#### 5.2 Preparation, Presentation & Relevance

An lecturer’s teaching and lecture style can positively or negatively affect the overall evaluation of a course, even if only to a small degree [13], [17], [18]. Our data also show that, above all, the presentation of the material is closely connected to the overall evaluation – even more strongly than the relevance of the topic itself. Although advance preparation on the part of students also correlates significantly with the overall evaluation, this connection is slight in our study. It is assumed that the lectures which students find relevant will be evaluated more positively [19]. Since this perception is not directly connected with the actual course, it is also presumed that a bias variable is involved [20]. In contrast, the presentation of a lecture is primarily dependent on the lecturer [21]. 

#### 5.3 Observations over time

In the final weeks of 2011, a significantly lower evaluation of the course was seen in Block 2. Two lectures were particularly criticized by the students in this block. It is assumed that the evaluations of these two lectures are primarily responsible for the discrepancy seen between the two blocks. With ARS, poorly rated lectures can be identified during the lecture phase and action can be taken in a very timely manner, for instance in the form of lecturer coaching or revision of the pertinent lecture content. With an end-of-term evaluation, this kind of action is much more limited.

#### 5.4 Overall evaluation, including a comparison with the end-of-semester evaluation

The lecture course on Pediatrics during the 2011-12 winter semester at the University of Freiburg Medical Center (Universitätsklinikum Freiburg) was evaluated on the end-of-semester evaluation with the score of 2.45. The same course received a score of 2.31 on the ARS evaluation. There is a statistically significant difference between these evaluation results. The inherent effect of ARS to lead to a more positive evaluation may be one of the decisive factors determining this outcome [9], [10], [11], [12]. 

#### 5.5 Feedback for the lecturers

Following the evaluation, the results were presented to the lecturers in an anonymized form. The lecturers could identify the results for their particular lecture based on an assigned number known only to them. The lectures were then subject to an internal review and revised as needed. All of the lecturers were offered training in lecture presentation. A repeat evaluation following these changes has yet to be done; the initial responses of the lecturers and students are positive. Overall, the feedback from lecturers regarding the rapid availability of the evaluation results was very positive. ARS was judged to be a suitable evaluation medium.

## 6. Conclusion

With little effort, lectures can be evaluated with ARS while they are taking place or immediately afterward. In addition, ARS can be used to make lectures interactive [5]. An important advantage of using ARS to evaluate educational courses is the immediate access to lecturer-specific feedback. This study was able to demonstrate that, by using ARS as an evaluation medium during or after a course, the variables of student preparation, lecture presentation, and relevance of the topic correlate significantly with the overall evaluation of a course.

Individual lectures that are given poor evaluations can lead to a lower evaluation for the course overall. However, this situation is immediately visible in the ARS results, so that timely intervention can take place, for instance in the form of special coaching for the affected lecturer. When relying solely on end-of-term evaluations, this is not possible.

A limitation of this study is that the number of students present in comparison with the number of students actually participating in the ARS evaluation was not determined.

Due to the advantages listed above, evaluations using ARS should be made available to other subjects following further standardization and refinement.

## Acknowledgement

We wish to thank all of the lecturers at the *Zentrum für Kinder- und Jugendmedizin Freiburg* and all of the participating students.

## Competing interests

The authors declare that they have no competing interests.

## Figures and Tables

**Table 1:**
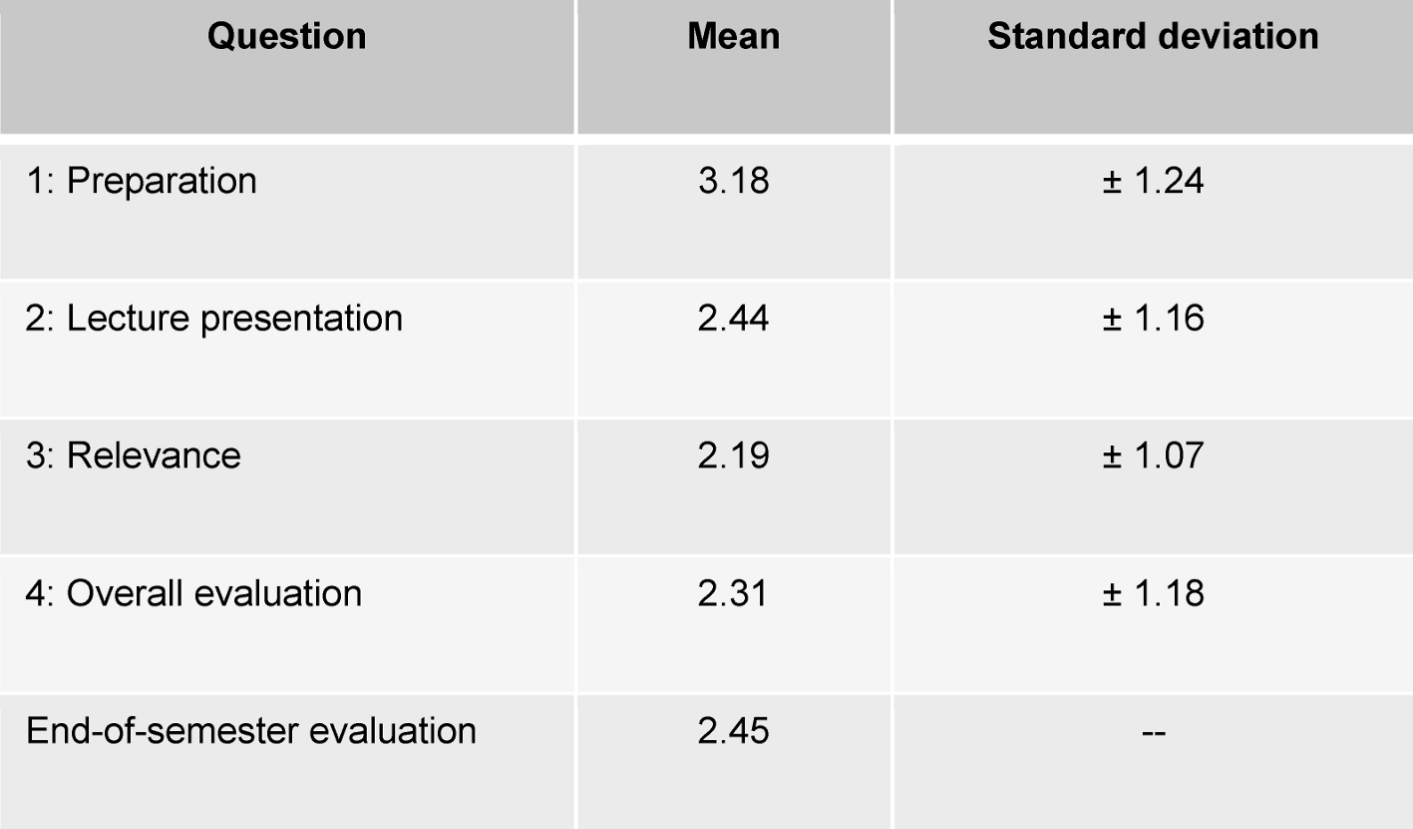
Means and standard deviation of the evaluation using ARS and mean of the online, end-of-term evaluation (standard deviation not available).

**Table 2:**
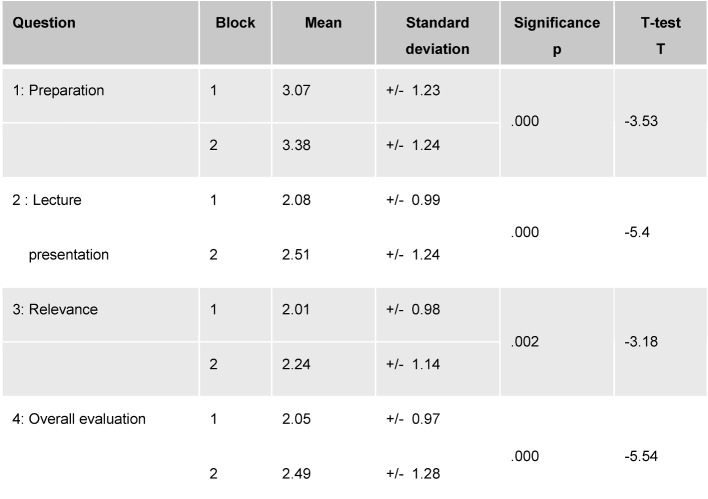
Comparison of the evaluation results for Block 1 (343≤N≤370) and Block 2 (405≤N≤463). The same lectures were offered in both blocks.

**Table 3:**
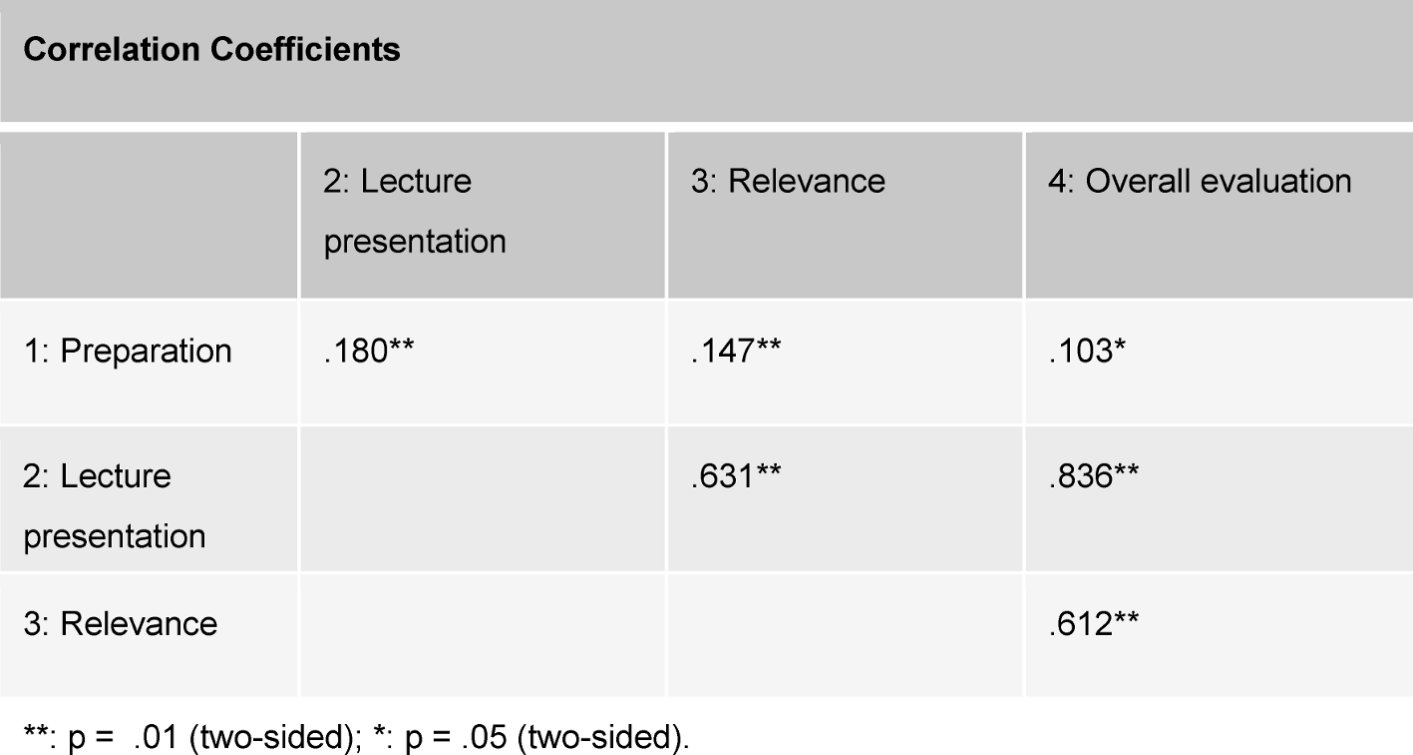
Results of the correlation analysis (based on Pearson) Number of responses per comparison: 541-810.
